# Exudation of organic acid anions by tropical grasses in response to low phosphorus availability

**DOI:** 10.1038/s41598-020-73398-1

**Published:** 2020-10-12

**Authors:** Danilo Silva Almeida, Lucas Benes Delai, Alexandra Christine Helena Frankland Sawaya, Ciro Antonio Rosolem

**Affiliations:** 1 Department of Crop Science, College of Agricultural Sciences, São Paulo State University, Botucatu, 18610-307 Brazil; 2 Faculty of Pharmaceutical Science, University of Campinas, Campinas, 13083-970 Brazil

**Keywords:** Plant ecology, Abiotic

## Abstract

It has been suggested that some tropical grasses can acquire phosphorus (P) from hematite and gypsite by exuding organic acid anions (OAs). However, it remains to be determined exactly which OAs could be involved in each case. The objective of this study was to verify the exudation OAs by ruzigrass (*Urochloa ruziziensis*), palisade grass (*U. brizantha*), and Guinea grass (*Megathyrsus maximus*) as a response to P deficiency. The grasses were grown in leachate columns with adequate and deficient P nutrient solutions. The concentration of OAs in the leacheate and root surface, as well as shoot and root dry matter, and P uptake were determined. Citrate, isocitrate, and malate concentration in leachates and root surfaces increased with P starvation, mainly for the Urochloa grasses. Oxalate exudation was similar for the grasses under adequate P supply, but was lower in Guinea grass under P starvation. Palisade grass showed a higher concentration of total OAs in the root surface than the other species due to a great production of oxalate and isocitrate. Palisade grass showed greater dry matter yields regardless of P deficiency, and Guinea grass always had the higher shoot:root ratio. *Urochloa* grasses have a higher capacity to cope with low P availability by exuding OAs along with a lower shoot:root ratio than Guinea grass.

## Introduction

Soil phosphorus (P) deficiency is one of the main factors limiting crop yield in acid and highly weathered tropical soils, where there is a predominance of kaolinite minerals, as well as iron (Fe) and aluminum (Al) oxides^1^. Only about 0.1% of the total P is in the soil solution, which is readily available to plants^2^. This typically low concentration of P in the soil solution is due to chemisorption reactions with 1:1 clay minerals, iron (Fe) and aluminum (Al) oxides and hydroxides, and precipitation reaction with Fe, Al and calcium (Ca) phosphates^3^. As a result, P availability to plants is limited by the supply rate from the soil solid phase to soil solution. However, plants have evolved several strategies to deal with low soil P availability^4^, including the exploration of a greater soil volume by changing root length and architecture^5^; the differential allocation of root biomass in soil layers where there is a higher P concentration^6^; the bio-stimulation and protocooperation with rhizosphere microorganisms that have P mobilization traits; the increase of P concentration in the soil solution through the root exudation of chemically active organic compounds into the rhizosphere; and an increase of P use efficiency by modifying their own physiology of tissue P allocation and use. More specifically, to increase the concentration of soil solution P, some plants exude organic acid anions (OAs), such as citrate, oxalate, and malate, and also enzymes, such as phosphatases into the rhizosphere^4,7^.

Several studies have shown at least two mechanisms through which exudation of OAs affects P release from the soil solid phase: (a) the direct competition of OAs with P for the adsorption sites on the surface of Fe and Al oxides, inducing P desorption^7,8^; and (b) the complexation of metal cations by OAs, inducing the solubilization of precipitated P^9,10^. The exudation of OAs also stimulates microbial activity in the rhizosphere, which contributes to increased P availability through scavenging recalcitrant P forms, such as inositol phosphates^11–13^.

The identification of plant species with the ability to acquire recalcitrant soil P forms has been proposed for the sustainable management of crop systems, specifically through their use as cover crops, to strategically promote soil P cycling and availability to the subsequent cash-crop in rotation^14^. Furthermore, the distinction and understanding of the adaptation mechanisms to low P soils by such species is important in the development of these technologies^15,16^. The use of tropical grasses as cover crops has been recently proposed to induce soil P cycling and to increase soil P availability to soybean^17^, specifically the grasses of the *Urochloa* genus (syn. *Brachiaria*), which are highly adapted to low fertility tropical soils^18^, while other species have been used as cover crop mainly due to a high biomass yield, such as Guinea grass (*Megathyrsus maximus*). Ruzigrass (*Urochloa ruziziensis*) and palisade grass (*U. brizantha*) can mobilize and take up recalcitrant P bound to Fe and Al^19^, and it has been suggested that their high P acquisition efficiency is due to OAs exudation^20^. However, there have been no studies confirming that OAs exuded by *Urochloa* grasses is one of the response mechanisms to P deficiency; likewise, there is no evidence that the higher demand of P fertilizer by Guinea grass^20^ is due to its low exudation of OAs. Therefore, the objective of this study was to evaluate the exudation of OAs by Urochloa grasses and Guinea grass in response to low P . Specifically, we aimed to test the hypothesis that the exudation of OAs by *Urochloa* grasses is higher under P deficiency, what has been reported as a mechanism to cope with low P soils, as compared with Guinea grass, which is less tolerant to P deficiency.

## Material and methods

### Experimental design

Three tropical grass species were grown in leachate columns following the method described by Tian et al.^21^ with modifications to allow sampling of OAs exuded by the roots in response to P levels supplied through nutrient solution. Seeds of ruzigrass [*Urochloa ruziziensis* (R. Germ. and C.M. Evrard) Morrone and Zuloaga], palisade grass [*U. brizantha* (A. Rich.) R.D. Webster], and Guinea grass [*Megathyrsus* *maximus* (Jacq.) B.K. Simon & Jacobs], were disinfected for 10 min in 2% sodium hypochlorite solution before being germinated and grown for 10 d in growth chambers, with a day/night cycle of 16 h of light at 35 °C and 8 h of darkness at 20 °C, on filter paper saturated with deionized water. Then, 10 uniform seedlings were selected and transplanted in 80-mL growth columns (3.6 cm in diameter at the upper base, 1.2 cm in diameter at the bottom base and 18 cm in height), filled with 90 g of dry inert sand, and placed in a greenhouse. The sand was prepared by washing with 0.1 mol L^−1^ hydrochloric acid solution, and then with deionized water to remove organic materials and clay. Then, the sand was sieved through a 1-mm sieve and air-dried. Each growth column was prepared by inserting a fiberglass net at the bottom to prevent loss of sand and to allow for leachate sampling. The sand water content was monitored daily, and deionized water was added carefully, avoiding loss of leachate. All growth columns were relocated every day during the study to reduce micro-environmental effects.

The three species were grown with high and low P leves in a complete randomized design, with five replications. Treatments with high and low P levels were established by adjusting the nutrient solution^22^ to 1.0 and 0.1 mmol L^−1^ KH_2_PO_4_, respectively. The concentration of 1.0 mmol L^−1^ KH_2_PO_4_ provides the typical P sufficiency concentration in Hoagland solution, while the 0.1 mmol L^−1^ KH_2_PO_4_ was used to induce P deficiency. To balance the K concentration in the treatment with low P level, 0.9 mmol L^−1^ KCl was used in the nutrient solution. The Hoagland solution also contained: 5.0 mmol L^−1^ Ca(NO_3_)_2_, 5.0 mmol L^−1^ KNO_3,_ 2.0 mmol L^−1^ MgSO_4_, 45.3 µmol L^−1^ H_3_BO_3_, 9.1 µmol L^−1^ MnSO_4_·H_2_O, 0.7 µmol L^−1^ ZnSO_4_·7H_2_O, 0.4 µmol L^−1^ CuSO_4_·5H_2_O, 0.1 µmol L^−1^ NaMoO_4_, and 89.2 µmol L^−1^ FeEDTA. The nutrient solution was applied as follows: Five days after the seedlings were first transplanted to the growth column and at 5-day intervals thereafter, 50 mL of nutrient solution was applied in each growth column.

The first leachate sample was collected 20 days after transplant, followed by four more collections at 5-day intervals. Therefore, the first and last samplings were performed 30 and 50 days after germination, respectively. To collect the leachate, the water holding capacity of the sand (25 mL of deionized water) was applied twice (50 mL) to ensure a complete displacement of the pore water, and the dripping leachate was collected in a 50 mL sample vial. Then, the leachate samples were sterile filtered through a 0.45-µm membrane and stored at – 18 °C for analysis.

After the last leachate sample collection, plants were harvested, roots were separated from shoots, placed in vials with 40 mL deionized water, and sonicated for 15 min to extract OAs from the root surface^23^. An ultrasonic bath was used in frequency of 40 kHz. After the removal of roots from the vials, the solution was sterile-filtered through a 0.45-µm membrane and stored at – 18 °C for analysis. According to Barber and Gunn^23^, sonication is a valid procedure for removing exudate from the root surface, and the structure of root hairs, protoplasmic straming, and subsequent uptake of phosphate are not affected by this treatment. Root and shoot samples were dried to a constant mass in an air-forced oven (65 °C), dry mass was evaluated, and then the samples were ground and sieved through a 1-mm screen for P analysis. The ground samples were submitted to double acid wet digestion, using a nitric and perchloric acid solution (HNO_3_ + HClO_4_, at a ratio of 2:1, v/v), and P concentration was determined by the vanadomolybdate method^24^.

### Organic acid anion quantification

The collected leachate samples and the sonicated extracts were lyophilized for approximately 48 h, until complete dryness. The residue was reconstituted in 1 mL of 0.1% (v/v) Mili-Q water-solved formic acid (pH = 5.8) to avoid microbial consumption of the organic acids, and was used for the quantification of the concentration of citrate, isocitrate, fumarate, lactate, malate, maleate, malonate, oxalate, succinate, and tartrate, in each leachate sample and in the root extracts^25^. Reconstituted samples were analyzed using an ultra-high-performance liquid chromatography system with a triple quadrupole mass spectrometer (UHPLC-MS) and an electrospray ionization source, Acquity UPLC-TQD (Waters, Milford, MA, U.S.). A Waters Acquity BEH C8 column (2.1 × 50 mm) with 1.7-μm particle size at a temperature of 30ºC was used to separate the compounds. The mobile phase consisted of Mili-Q water containing 0.1% (v/v) of formic acid (phase A), and methanol (phase B) at a flow-rate of 0.2 mL min^−1^. The injected volume was 10 μL. Elution was performed using a gradient starting with 99% A, which was maintained for 2.5 min, then it was ramped up to 50% A at 3 min, this concentration was kept up to 4 min, and then was returned to the initial conditions and the system was re-equilibrated until 6 min. The MassLynxV4 software was used to acquire and process all data. For the specific detection of analytes it was used MS detection in the negative ion mode and Selected Ion Mode. The source and desolvation temperatures were 150 °C and 350 °C, respectively. Capillary voltage was − 2.8 kV and cone − 25 V. Identification of OA was done by comparing retention times and mass spectra with known standards. The data of each leachate sampling time was used to calculate the average concentration of OA in the leachates for each replicate of each treatment.

### Statistical analysis

The results were subjected to a 2-way ANOVA considering a 3 species × 2 P levels factorial in a complete randomized design, using a general linear model (Proc GLM) in SAS software (version 9.4, SAS Inst., North Carolina, U.S.),. When the F test was significant (*p* < 0.05), treatment means were compared by Student’s t-test (*p* < 0.05).

## Results

### Concentration of organic acid anions exudated

The total concentration of OAs in leachates was higher under P deficiency than P sufficiency for ruzigrass and palisade grass (Table 1), and citric, oxalic and lactic acids, were, in this order, exuded in higher amounts by the tropical grasses (Table 1), irrespective of P supply. Although citrate and isocitrate are isomers, the retention time of these anions in the UPLC-MS was different. Among all the measured organic acids, the highest overall increase in exudation in response to P deficiency was observed for citrate for all three grass species. Ruzigrass showed the highest increase in citrate exudation with concentrations reaching 72.7 nmol L^−1^ under P deficiency, while the concentration was 20.5 nmol L^−1^ in P sufficiency (Table 1). The concentrations of isocitrate and malate were only affected by P supply for ruzigrass. The concentration of lactate was higher in response to P deficiency only for palisade grass, and was also higher than the exudation by the other species. The exudation of oxalate by Guinea grass was lower than by the other grasses under P starvation. Only the concentration of succinate was higher in P sufficiency than P deficiency in all three grasses.

**Table 1:** Concentration organic acid anions in leachates from sand, as affected by grass species and P supply.

P supply	Grass species		
	Ruzi grass	Palisade grass	Guinea grass
	Citrate (nmol L^−1^)		
Low P	72.7 Aa^a^	48.1 Ab	52.8 Ab
High P	20.5 Ba	14.6 Ba	17.0 Ba
	Isocitrate (nmol L^−1^)		
Low P	7.03 Aa	4.82 Aa	5.30 Aa
High P	2.94 Ba	5.13 Aa	6.21 Aa
	Fumarate/maleate (nmol L^-1^)		
Low P	3.93^ns^	4.12^ns^	3.17^ns^
High P	4.28^ns^	3.92^ns^	3.28^ns^
	Lactate (nmol L^−1^)		
Low P	27.1 Ab	48.9 Aa	22.7 Ab
High P	23.3 Aa	22.7 Ba	15.6 Aa
	Malate (nmol L^−1^)		
Low P	9.54 Aa	10.81 Aa	7.32 Aa
High P	2.74 Bb	7.14 Aa	6.07 Aa
	Oxalate (nmol L^−1^)		
Low P	46.7 Aa	40.8 Aa	25.4 Ab
High P	40.8 Aa	38.3 Aa	39.8 Aa
	Succinate (nmol L^−1^)		
Low P	16.8 Ba	20.0 Ba	20.1 Ba
High P	30.5 Aa	27.7 Aa	34.1 Aa
	Tartrate (nmol L^−1^)		
Low P	4.64^ns^	3.97^ns^	3.53^ns^
High P	3.58^ns^	3.45^ns^	3.59^ns^
	Total (nmol L^−1^)		
Low P	188 Aa	181 Aa	140 Ab
High P	129 Ba	123 Ba	126 Aa

^a^Averages followed by different lowercase letters in lines and uppercase in columns are significantly different (t-test, *p* < 0.05).

^ns^Not significant.

Fumarate and maleate anions are isomers and had the same retention time in the UPLC-MS. Therefore, it was not possible to quantify the concentration of each of these anions, and the results were interpreted as the sum of their concentrations. As well as tartrate, the concentration of fumarate/maleate in the leachates was not affected by the treatments (Table 1). The concentration of malonate was below the limit of detection.

### Concentration of organic acid anions in the root surface

While citrate was the organic anion exuded in highest concentration in the leachate for all grasses, isocitrate was the predominant form in the root surface of ruzigrass and palisade grass. The concentration of isocitrate was, on average, nearly 37 and 27 times greater than the citrate concentration in palisade grass and ruzigrass root surface, respectively (Table 2). For Guinea grass, citrate, malate, and lactate were the predominant organic anions in the roots. The concentration of citrate was similar in Guinea and palisade grass, and higher than in ruzigrass root surface. However, the concentration of isocitrate in palisade grass and ruzigrass was much higher than in Guinea grass.

**Table 2:** Concentration of organic acid anions on the root surface as affected by grass species and P supply.

P supply	Grass species		
	Ruzi grass	Palisade grass	Guinea grass
	Citrate (nmol g^−1^ dry mass)		
Low P	38 Ab^a^	132 Aa	162 Aa
High P	24 Ab	51 Bab	63 Ba
	Isocitrate (nmol g^−1^ dry mass)		
Low P	1667 Ab	4061 Aa	18 Ac
High P	650 Ba	794 Ba	15 Ab
	Fumarate/maleate (nmol g^−1^ dry mass)		
Low P	0.018 Aa	0.010 Aa	0.018 Aa
High P	0.004 Ba	0.006 Aa	0.012 Aa
	Lactate (nmol g^−1^ dry mass)		
Low P	57 Ab	10 Bc	119 Aa
High P	79 Aa	78 Aa	98 Aa
	Malate (nmol g^−1^ dry mass )		
Low P	8 Bc	366 Aa	146 Ab
High P	98 Ab	190 Ba	272 Aa
	Oxalate (nmol g^−1^ dry mass )		
Low P	65 Ab	1143 Ba	66 Ab
High P	83 Ab	1584 Aa	75 Ab
	Succinate (nmol g^−1^ dry mass)		
Low P	4.81 Bb	4.94 Ab	9.61 Aa
High P	8.41 Aa	7.07 Aa	8.70 Aa
	Tartrate (nmol g^−1^ dry mass )		
Low P	1.39^ns^	0.41^ns^	1.54^ns^
High P	0.51^ns^	0.51^ns^	1.30^ns^
	Total (nmol g^−1^ dry mass )		
Low P	1841 Ab	5717 Aa	523 Ab
High P	943 Bb	2704 Ba	534 Ab

^a^Averages followed by different lowercase letters in lines and uppercase in columns are significantly different (t-test, *p* < 0.05).

^ns^Not significant.

The concentration of fumarate/maleate in the root surface of the grasses was lower than the concentration of the other organic anions, and was not affected by grass species. It was not possible to evaluate the malonate concentration, as it was below quantification levels in the samples. The concentration of lactate was higher in Guinea grass than in ruzigrass and palisade grass roots under P starvation. The concentration of malate was higher in ruzigrass growing in P sufficiency than under P deficiency, while the opposite was observed in palisade grass roots. The concentration of oxalate in palisade grass roots was about 18 times greater than in the other species. Only ruzigrass showed higher succinate concentration on the root surface when P supply was high. The concentration of tartrate in the grass roots was not affected by the treatments (Table 2).

The total organic anions concentration in palisade grass roots was greatest compared with the other species (Table 2). The Urochloa species had a great concentration of organic anions in the roots when P supply was low, while the concentration in Guinea grass roots was not affected by P supply.

### Dry matter yield and phosphorus uptake

The shoot dry matter yield of palisade grass was higher than that of ruzigrass when grown under P deficiency (Table 3). When P supply was high, the dry matter of Guinea grass shoot was higher than ruzigrass. The root dry matter of palisade grass was higher than ruzigrass, which in turn was higher than that of Guinea grass, at high P supply. Under P deficiency, palisade grass total dry matter yield was greater than that of the other species. However, when P supply was high, total dry matter of palisade grass was only higher than ruzigrass. Guinea grass showed the highest shoot:root ratio, while the significantly greater root production of palisade grass resulted in the lowest shoot:root ratio among the species (Table 3).

**Table 3:** Grass shoot, root, and total dry matter, shoot:root ratio, and P uptake in grass shoot, root, and total, as affected by grass species and P supply.

P supply	Grass especies		
	Ruzi grass	Palisade grass	Guinea grass
	Shoot dry matter (g pot^-1^)		
Low P	2.31 Bb^a^	3.05 Ba	2.68 Bab
High P	5.05 Ab	5.06 Aab	5.36 Aa
	Root dry matter (g pot^-1^)		
Low P	0.63 Bb	1.19 Ba	0.60 Bb
High P	1.49 Ab	1.86 Aa	1.22 Ac
	Total dry matter (g pot^-1^)		
Low P	2.94 Bb	4.23 Ba	3.29 Bb
High P	6.54 Ab	6.92 Aa	6.58 Aab
	Shoot:root ratio (g g^-1^)		
Low P	3.66 Ab	2.59 Ac	4.46 Aa
High P	3.39 Ab	2.75 Ac	4.44 Aa
	Shoot P uptake (mg pot^-1^)		
Low P	0.87 Ba	1.16 Ba	1.13 Ba
High P	6.00 Ab	6.62 Aab	7.00 Aa
	Root P uptake (mg pot^-1^)		
Low P	0.38 Bb	0.69 Ba	0.35 Bb
High P	2.03 Aa	1.72 Ab	1.70 Ab
	Total P uptake (mg pot^-1^)		
Low P	1.25 Ba	1.84 Ba	1.48 Ba
High P	8.03 Aa	8.35 Aa	8.70 Aa

^a^Averages followed by different lowercase letters in lines and uppercase in columns are significantly different (t-test, *p* < 0.05).

^ns^Not significant.

Greater shoot P uptake was observed for Guinea grass than ruzigrass when P supply was low. Root P uptake by palisade grass was higher than for the other species under low P availability, but when P supply was high a greater P uptake was observed for ruzigrass compared with the other grasses. However, the total P uptake was not different between grasses, regardless of P supply (Table 3).

## Discussion

Several root exudate components have been identified in the rhizosphere of different species growing under low P availability, including all the OAs evaluated in this study^11,26^. The production of citrate, isocitrate and malate quantified both in the leachates and in the root surface was an indication of the grass response to low P availability. To date, this is the first report of changes in the exuded OAs by roots of palisade grass and Guinea grass in response to low P supply. In a recent study it has been reported the exudation of acetate, formate, glycolate, lactate, and oxalate from ruzigrass and signal grass (*Urochloa decumbens* Stapf cv. Basilisk) under low P supply, but not the exudation of citrate^27^, which was observed here as the main response to P starvation. The accumulation of citrate, malate, and oxalate in ruzigrass and signal grass roots in response to Al toxicity has been also reported^28^. Few studies have estimated the composition of OAs in the rhizosphere or in the root surface of palisade grass. Citrate, acetate, lactate, and small amounts of oxalate were found in the rhizosphere of palisade grass^29^, and high concentrations of citrate, malate, and aspartate were observed in palisade grass roots and leaves under high Al levels^30^.

Interestingly, the concentration of isocitrate was much greater than citrate on the root surface. Isocitrate is a structural isomer of citrate and is formed after conversion of citrate by aconitase in the cytosol^31^. Citrate exudation by roots has been extensively studied, while isocitrate production is usually ignored. Citrate is a tri-carboxylic anion and is recognized as one of the most effective OAs for increasing available P in soils^9^. The arrangement of the hydroxyl groups in citrate results in a high capacity to complex metals and compete with P for adsorption sites^7^. Since citrate and isocitrate are isomers, they are expected to have similar roles in improving soil P availability. Nevertheless, the greater amount of isocitrate was observed on the root surface of ruzigrass and palisade grass, whereas citrate was the main OA present in the leachates, suggesting that isocitrate may be a precursor of the citrate found in the nutrient solution. Further studies are needed to evaluate the relationship between *in-planta* isocitrate production and the citrate exudation.

Other OAs often studied in P starved plants, such as oxalate and malate^11^ were also observed in large concentrations in this study, mainly in palisade grass root surface. Oxalate has a high capacity to release P from Ca-phosphate minerals, which could improve the solubilization of reactive rock phosphates^16^, while citrate and malate may release P mainly held in Fe- and Al-phosphate, due to a greater affinity for Fe and Al^32^.

Despite fumarate being reported in great concentration in the rhizosphere of other species adapted to low fertile soils, such as coast banksia (*Banksia integrifolia* L.f.)^33^, in our study the tropical grasses showed just trace concentrations of fumarate and maleate, and no malonate was found in the root surface. Malonate production has been reported in pigeon pea [*Cajanus cajan* (L.) Huth], a C3- type photosynthesis species adapted to low P soils^34^. Fumarate, maleate, lactate, succinate, and tartrate are usually found in low concentrations in plants^26^, and at least fumarate, maleate and lactate, have a low ability to form stable complexes with cations^37^. So, these are not expected to cause significant direct P solubilization, but may instead act as a microbial stimulant, causing an indirect effect on P availability^13,36^.

The characterization of below-ground traits such as OAs exudation is often difficult due to *in-situ* degradation, spatial and temporal heterogeneities and the usual low concentration in exudation extracts^11^. Nevertheless, the leachate sampling method used in this study allowed for new insights into the evaluation of OAs exuded by grasses in a simple way, and in an environment more similar to the natural condition than the usual experimental setup using deep water culture hydroponic systems^38,39^. Growing grasses in pure sand, instead of soil with clay particles, reduced the effect of chemisorption reactions and reduced microbial degradation allowing for a better quantitative assessment of OA concentration in leachate samples. In the absence of soil colloids, even organic acid metal complexes have the potential to leach^39^. It has been clearly demonstrated that microorganisms degrade OAs, drastically reducing their concentration in soils, and most of the OAs effect on P mobilization may be indirect through stimulation of the microbial community, rather than a direct chemical effect^13,36^.

Most studies have applied OAs in a concentration much higher than those observed here to show the mechanism of competition betwenn OAs and P for adsorption sites in soils^9,10^. Other authors, however, consider that the continuous exudation of OAs may render a cumulative effect on the competition with soil P for adsorption sites even at low exudation rates^35^. Both P mobilization and microbial stimulation are known to be important factors to improve soil P availability to plants^26^; however, despite this subject having been widely explored, there is still no consensus on whether OAs directly mobilize P from soil solid phase into soil solution or act indirectly by feeding the microbial community in the rhizosphere. Our study shows a clear OA exudation response to P starvation, but does not address the issue of the mechanisms by which these OAs function in mobilizing soil P.

The lower total OA concentration in Guinea grass, as well as the lack of response of this species to the low P supply is evidence that OAs production and exudation may not be a mechanism of this species to deal with low P soils. Thus, the high fertilizer P demand reported for Guinea grass^20^ may be a result of the lack of rhizosphere P mobilization traits. On the other hand, ruzigrass and palisade grass seems to be much more adapted to low P environments, showing a higher concentration of OAs compared with Guinea grass, as verified in the leachate samples, and showing twice the concentration of OAs on the root surface under low P.

The greater adaptation of *Urochloa* grasses than Guinea grass to low P soils^20^ seems to be related not only to the higher OA production, but also to other mechanisms, such as the ability to change the shoot:root ratio. A shift in biomass partitioning towards the root system was not observed in response to P starvation in the present experiment. However, there was a great difference in shoot:root ratio among the grasses, which may be an indication of the ability of each species to cope with low P soils. It has been identified the accumulation of 1,3-di-*O*-*trans*-feruloylquinic acid (DFQA) in ruzigrass roots^37^, which can be linked to preferential biomass allocation to roots. Palisade grass seems to have an even higher capacity to produce roots than ruzigrass, and this is not only related to soil P level, as shown in this study. The greater capacity of palisade grass than ruzigrass to produce roots has been observed at high Al levels^41^, which emphasizes the high adaptation of this species to low fertility soils. In a *Urochloa* hybrid of ruzigrass and palisade grass, the effect of low P supply on a greater carbon allocation to roots has been observed^40^. For the *Urochloa* hybrid, a smaller proportion of C allocated in sugars and a larger proportion of C destined to OAs, as well as a great ability to use P efficiently enhancing P recycling has been observed in comparison with rice (*Oriza sativa* L.)^42^. Additionally, a low shoot internal requirement for P and a high P uptake efficiency per unit of root length of ruzigrass than coronivia grass [*Urochloa dyctioneura* (Fig. and De Not.) Veldkamp] were reported^43^.

Considering that *Urochloa* grasses showed a higher ability to cope with low P soils due to the greater carbon allocation to roots and higher OAs exudation, a higher total P uptake would be expected compared with Guinea grass. However, in this experiment, P was supplied as a highly available form (KH_2_PO_4_), using the Hoagland nutrient solution differing only in the P concentration according to each treatment, which was enough to simulate a low and high soil P availability. Therefore, there was P readily available in the solution, which may have prevented a response in dry matter yields. Nevertheless, the OAs results may be a good indication that *Urochloa* grasses, mainly palisade grass, are a better option to be grown in low P soils. The introduction of these grasses in rotation with cash crops has been suggested^17^ and may result in an increased cycling of recalcitrant P forms, and eventually in a better use of the residual P fertilizers from previous years, an essential step to a more sustainable future for agriculture.

## Conclusions

Palisade grass and ruzigrass exude more total OAs and more oxalate than Guinea grass under low-P conditions. The root exudation of isocitrate and oxalate by *Urochloa* grasses in response to P starvation seems to be a tolerance mechanism. Additionally, the higher adaptation of *Urochloa* grasses compared with Guinea grass to low P availability is also due to a greater relative carbon allocation into roots, resulting in lower shoot:root ratio. The lack of soil P mobilizing mechanisms in Guinea grass may be a reason for its known higher demand for P fertilizer than ruzigrass and palisade grass.
